# Association between peripheral and coronary microvascular function and the impact of myocardial bridging

**DOI:** 10.14814/phy2.70340

**Published:** 2025-04-23

**Authors:** Takumi Toya, Shotaro Yoshida, Kazuhiko Kuinose, Shun Akai, Tsukasa Urushima, Kaoru Naito, Masanori Oobori, Yusei Nakashima, Akira Miyauchi, Yuji Nagatomo, Takeshi Adachi

**Affiliations:** ^1^ Division of Cardiology National Defense Medical College Saitama Japan; ^2^ Department of Cardiology NHO Tokyo Medical Center Tokyo Japan; ^3^ School of Medicine National Defense Medical College Saitama Japan

**Keywords:** coronary microvascular dysfunction, myocardial bridging, peripheral microvascular dysfunction

## Abstract

The reactive hyperemia index (RHI) is a marker of peripheral microvascular function influenced by both endothelium‐dependent and independent mechanisms. Myocardial bridging (MB) may impact coronary microvascular function, but its effect on the relationship between RHI and coronary microvascular function remains unclear. In this cross‐sectional study, 38 patients underwent noninvasive RHI assessment alongside invasive coronary microvascular function testing. MB was identified via coronary angiography, while endothelium‐dependent and independent coronary microvascular function were evaluated using coronary flow reserve in response to intracoronary administration of acetylcholine and microvascular resistance reserve in response to intravenous administration of adenosine, respectively. Among 38 patients (mean age: 59 years, 34% male), 14 had MB. RHI correlated with an index of endothelium‐independent coronary microvascular function (*r* = 0.34, *p* = 0.04), whereas it did not correlate with that of endothelium‐dependent function. This correlation persisted in patients without MB (*r* = 0.54, *p* = 0.01) but was lost in those with MB (*p* = 0.83). RHI is associated with endothelium‐independent coronary microvascular function, but MB disrupts this relationship, suggesting a local impact on coronary microvascular physiology.

## 1 INTRODUCTION

Vascular disease is widely recognized as a systemic condition, enabling the assessment of remote vascular function through peripheral vascular measurements (Bonetti et al., 2003, 2004; Lerman & Zeiher, 2005). The reactive hyperemia index (RHI), derived from digital arterial measurements, serves as a marker of peripheral microvascular function. Reduced RHI has been associated with accelerated aging, heightened cardiovascular risk, cerebrovascular events, and malignancies, reflecting systemic microvascular pathology (Toya, Ahmad, et al., 2021; Toya, Sara, Ahmad, et al., 2020; Toya, Sara, et al., 2021; Toya, Sara, Corban, et al., 2020; Toya, Sara, Lerman, et al., 2020). In contrast, flow‐mediated dilation (FMD) of the brachial artery is a marker of macrovascular function. While both RHI and FMD assess post‐ischemic reactive hyperemia, they reflect distinct vascular domains: FMD is specific to conduit arteries (macrovessel), whereas RHI pertains to resistance arteries (microvessel) (Tanaka et al., 2018).

The mechanisms regulating FMD and RHI also differ significantly (Hamburg et al., 2011; Schnabel et al., 2011). FMD is predominantly governed by an endothelium‐dependent pathway mediated by nitric oxide (NO) (Joannides et al., 1995), and its strong association with coronary endothelial function underscores its diagnostic utility (Anderson et al., 1995; Broxterman et al., 2019). In contrast, RHI reflects a combination of endothelium‐dependent (approximately 50% NO‐mediated) and endothelium‐independent mechanisms (Nohria et al., 2006). Similarly, coronary microvascular function is regulated through both endothelium‐dependent and independent pathways. However, data regarding the correlation between RHI and these distinct aspects of coronary microvascular function remain limited.

Myocardial bridging (MB), a tunneled segment of epicardial coronary arteries within the myocardium that has been traditionally regarded as a benign anatomical variant, has gained attention for its potential role in ischemia with nonobstructive coronary artery disease (INOCA) (Toya, 2025). MB‐induced disruption of coronary wall shear stress has been implicated in epicardial endothelial dysfunction (Corban et al., 2014), while impaired regulation of vasomotor substances such as endothelin‐1 may contribute to coronary microvascular dysfunction (Sara et al., 2020).

Given the systemic nature of microvascular disease, a correlation between RHI and coronary microvascular function would be expected. This study aimed to investigate the relationship between RHI and endothelium‐dependent and independent coronary microvascular function. Additionally, we sought to explore the impact of MB, which can locally alter coronary vasomotor function, on the association between peripheral and coronary microvascular function.

## 2 METHODS

### 2.1 Study population

In this cross‐sectional study, we enrolled 38 patients who underwent non‐invasive peripheral microvascular function assessment using the EndoPAT 2000 device (Itamar Medical Inc., Caesarea, Israel) and invasive coronary microvascular function testing on the same day at our center between August 2023 and December 2024. These evaluations were performed to investigate the cause of chest pain or discomfort in the absence of significant obstructive coronary artery disease. The study adhered to the principles outlined in the Declaration of Helsinki and received approval from the National Defense Medical College ethics committee (#4803). Written informed consent was obtained from all participants prior to their inclusion in the study.

### 2.2 Assessment of peripheral microvascular endothelial function

The RHI was employed to assess peripheral microvascular function, as described in the prior studies (Toya, Ahmad, et al., 2021; Toya, Sara, Ahmad, et al., 2020; Toya, Sara, et al., 2021; Toya, Sara, Corban, et al., 2020; Toya, Sara, Lerman, et al., 2020). The protocol consisted of a 5‐min baseline measurement, followed by 5 min of blood pressure cuff inflation on the test arm to 60 mmHg above baseline systolic blood pressure (up to a maximum of 200 mmHg). This was followed by a 5‐min measurement of peripheral arterial tonometry after cuff deflation. The contralateral arm served as a control without cuff occlusion. The reactive hyperemia peripheral arterial tonometry ratio was calculated as the average pulse wave amplitude during the 1‐minute period starting 1 min after cuff deflation (test arm = A; control arm = C), divided by the average baseline pulse wave amplitude during the preceding 3.5 min (test arm = B; control arm = D). The RHI was automatically computed by normalizing the baseline signal and indexing the test arm ratio to the control arm (RHI = [A/B]/[C/D] × [baseline correction]). Per protocol, patients discontinued vasoactive medications, including calcium channel blockers, β‐blockers, and long‐acting nitrates, at least 24 h before testing. Participants fasted for 4 h prior to the study and refrained from caffeine and tobacco consumption on the day of the RHI assessment. An RHI value <2 was considered indicative of peripheral microvascular dysfunction (Borlaug et al., 2010; Taher, Sara, Toya, Borlaug, et al., 2020; Taher, Sara, Toya, Shepherd, et al., 2020; Toya, Ahmad, et al., 2021; Toya, Sara, Ahmad, et al., 2020; Toya, Sara, Corban, et al., 2020).

### 2.3 Coronary angiography and assessment of myocardial bridging

All diagnostic coronary angiography procedures were performed by interventional cardiologists following the standard institutional guidelines. A transfemoral or transradial approach was utilized, with at least four angiographic views of the left anterior descending artery (LAD) obtained after intracoronary nitroglycerin administration. MB was identified visually during coronary angiography by observing transient constriction or a “milking effect” of the LAD between systole and diastole, as previously described (Mahmoudi Hamidabad et al., 2024; Sara et al., 2020). At least two investigators independently reviewed angiographic images to determine the presence or absence of dynamic compression in the LAD, with consensus reached in cases of disagreement. To minimize bias, the “step‐down and step‐up” phenomenon—defined as a localized vessel trajectory change toward the ventricle—was not used as a diagnostic criterion. This precaution was taken to reduce the potential for overestimating dynamic compression, particularly in equivocal cases, where this phenomenon might influence observer interpretation (Kim et al., 2009).

### 2.4 Assessment of endothelium‐dependent and independent coronary microvascular function

The institutional protocol for invasive coronary microvascular function testing is as follows. Patients without significant epicardial coronary artery disease were selected for this assessment. A bolus of 5000 U of heparin (#22600AMX00809000, Mochida Pharmaceutical, Tokyo, Japan) was administered intravenously, followed by calibration and equalization of a 0.014‐inch pressure and temperature sensor‐equipped wire (PressureWire X, Abbott, IL, USA) to the 5‐6F guide catheter pressure. The wire was then advanced to the distal two‐thirds of the LAD.

Pressure and thermodilution measurements were performed at baseline and 3 min after the initiation of intravenous adenosine (#21700AMZ00057, Kowa, Aichi, Japan) infusion at 180 μg/kg/min to induce maximum hyperemia. Coronary flow reserve (CFR), index of microvascular resistance (IMR), and fractional flow reserve (FFR) were simultaneously assessed during hyperemia using commercially available software (CoroFlow, Abbott, IL, USA). CFR was calculated as the ratio of hyperemic blood flow (1/mean transit time [*T*mn], hyperemia) to resting blood flow (1/Tmn, resting). IMR was determined as the ratio of hyperemic mean Pd to hyperemic flow (Pd/[1/Tmn, hyperemia]). FFR was calculated as the ratio of hyperemic distal coronary pressure to hyperemic aortic pressure (Pd/Pa). Additionally, microvascular resistance reserve (MRR) was manually calculated using the formula: CFR/FFR×(Pa, resting)/(Pa, hyperemia), serving as a marker of endothelium‐independent coronary microvascular function (Toya & Lerman, 2023).

Following the evaluation of endothelium‐independent microvascular function, endothelium‐dependent coronary microvascular function was assessed using acetylcholine (Ach; # 22000AMX00795, Daiichi Sankyo, Tokyo, Japan). Pressure and thermodilution measurements were obtained at baseline and 3 min after intracoronary infusion of Ach (40 μg over 3 min) (Ahmad et al., 2023; Kanaji et al., 2024). Ach CFR, defined as the ratio of post‐Ach flow (1/*T*mn, Ach) to resting flow (1/*T*mn, resting), was used as a marker of endothelium‐dependent coronary microvascular function (Sinha et al., 2024).

### 2.5 Statistical analyses

Continuous variables distributed normally were expressed as mean ± SD, and those with a skewed distribution were expressed as the median with interquartile range. Categorical variables were expressed as frequency (percentage). To compare variables between groups, we performed an unpaired *t*‐test for normally distributed continuous variables, a Mann–Whitney *U* test for non‐normally distributed variables, and a chi‐squared test (or Fisher's exact test) for categorical variables. Linear regression analysis was performed to identify correlations between two parameters. The correlations between parameters were assessed using Pearson's or Spearman's correlation test as appropriate. For all tests, a two‐tailed *p* value <0.05 was considered statistically significant. All statistical analyses were performed using JMP Pro software (SAS Institute, Inc., NC, USA).

## 3 RESULTS

### 3.1 Baseline characteristics

Thirty‐eight patients successfully underwent both peripheral and coronary microvascular function testing. The baseline characteristics of the patients were summarized in Table 1. The cohort had a mean age of 59 years, with 34% being male. The prevalence of coronary risk factors was as follows: hypertension (61%), dyslipidemia (42%), and diabetes mellitus (13%). Approximately half of the patients exhibited peripheral microvascular dysfunction, defined as an RHI <2.0.

**TABLE 1:** Baseline characteristics of patients with and without myocardial bridging.

	Total (*N* = 38)	MB+ (*N* = 14)	MB− (*N* = 24)	*p*
Age (years)	59.3 ± 14.6	61.6 ± 18.3	58.1 ± 12.3	0.53
Female/Male (%)	13 (34)/25 (66)	4 (29)/10 (71)	9 (38)/15 (63)	0.58
Hypertension (%)	23 (61)	8 (57)	15 (63)	0.74
Dyslipidemia (%)	16 (42)	5 (36)	11 (46)	0.54
Diabetes mellitus (%)	5 (13)	2 (14)	3 (13)	0.88
Smoking (%)				
Never/former/current	14 (37)/16 (42)/8 (21)	6 (43)/6 (43)/2 (14)	8 (33)/10 (42)/6 (25)	0.70
History of coronary artery disease (%)	4 (11)	1 (7)	3 (13)	0.60
Family history of coronary artery disease (%)	5 (13)	2 (14)	3 (13)	0.88
Chronic kidney disease (%)	10 (26)	2 (14)	8 (33)	0.20
Body mass index (kg/m^2^)	24 ± 3.9	24.9 ± 4.0	23.5 ± 3.9	0.32
Ln RHI	0.72 ± 0.25	0.69 ± 0.26	0.75 ± 0.25	0.54
RHI <2	18 (47)	7 (50)	11 (46)	0.80

Abbreviations: Ln, log transformed; MB, myocardial bridging; RHI, reactive hyperemia index.

Among the 38 patients, 14 were identified with MB in the LAD, while the remaining 24 patients did not have MB in the LAD. The prevalence of coronary risk factors was comparable between those with and without MB. Additionally, peripheral microvascular function, as well as the proportion of patients with peripheral microvascular dysfunction, showed no significant differences between the two groups.

### 3.2 Coronary physiological parameters in patients with and without MB

Coronary physiological parameters are summarized in Table 2. Resting and hyperemic Pa and distal coronary pressure Pd were comparable between patients with and without MB, resulting in no significant difference in FFR between the groups. However, the IMR was significantly elevated in patients with MB compared to those without, despite similar values for other indicators of endothelium‐independent coronary microvascular function, including CFR and MRR.

**TABLE 2:** Coronary physiological parameters in patients with and without myocardial bridging.

	Total (*N* = 38)	MB+ (*N* = 14)	MB− (*N* = 24)	*p*
Resting Pa, mmHg (before adenosine test)	90.7 ± 13.6	93.4 ± 15.3	89.3 ± 12.5	0.39
Resting Pd, mmHg (before adenosine test)	85.9 ± 13.3	87.9 ± 14.3	84.7 ± 12.8	0.49
Hyperemic Pa (mmHg)	79.4 ± 14.1	82.7 ± 14.7	77.5 ± 13.7	0.29
Hyperemic Pd (mmHg)	71.7 ± 14.1	75.1 ± 13.0	69.7 ± 14.6	0.25
Fractional flow reserve	0.90 ± 0.07	0.90 ± 0.04	0.89 ± 0.08	0.76
Resting *T*mn (s) (before adenosine test)	0.96 (0.65, 1.34)	1.01 (0.74, 1.53)	0.91 (0.62, 1.31)	0.3
Hyperemic *T*mn (s)	0.28 (0.19, 0.36)	0.36 (0.26, 0.37)	0.25 (0.16, 0.32)	0.05
CFR	3.9 (2.9, 5.6)	3.3 (2.6, 5.2)	3.9 (2.9, 5.8)	0.27
IMR	20.4 (11.9, 25.3)	24.3 (19.6, 27.8)	17.9 (10.4, 22.8)	0.03
MRR	4.8 (3.7, 6.6)	4.6 (3.1, 6.3)	4.8 (3.9, 7.1)	0.3
Resting Pa, mmHg (before Ach test)	91.1 ± 15.0	92.5 ± 16.9	90.1 ± 14.0	0.68
Resting Pd, mmHg (before Ach test)	86.5 ± 14.0	87.2 ± 15.8	86.1 ± 13.0	0.85
Ach Pa (mmHg)	91.7 ± 12.9	94.2 ± 14.2	90.0 ± 12.0	0.4
Ach Pd (mmHg)	85.1 ± 13.5	84.8 ± 16.2	85.3 ± 11.7	0.93
Ach Pd/Pa	0.93 ± 0.07	0.90 ± 0.10	0.95 ± 0.03	0.11
Resting *T*mn, s (before Ach test)	0.90 (0.62, 1.23)	0.94 (0.77, 1.40)	0.86 (0.55, 1.18)	0.2
Ach *T*mn (s)	0.64 (0.50, 1.0)	0.54 (0.40, 1.12)	0.66 (0.53, 0.97)	0.62
Ach CFR	1.3 (1.1, 1.7)	1.6 (1.2, 2.4)	1.20 (0.9, 1.5)	0.02

Abbreviations: Ach, acetylcholine; CFR, coronary flow reserve; IMR, index of microvascular resistance; MB, myocardial bridging; MRR, microvascular resistance reserve; Pa, mean aortic pressure; Pd, mean distal coronary pressure; *T*mn, mean transit time.

Similarly, Pa and Pd measured before and after Ach administration did not differ significantly between the two groups. Notably, Ach CFR, an indicator of endothelium‐dependent coronary microvascular function, was significantly higher in patients with MB than in those without.

### 3.3 Correlation between peripheral and coronary microvascular function

**FIGURE 1:**
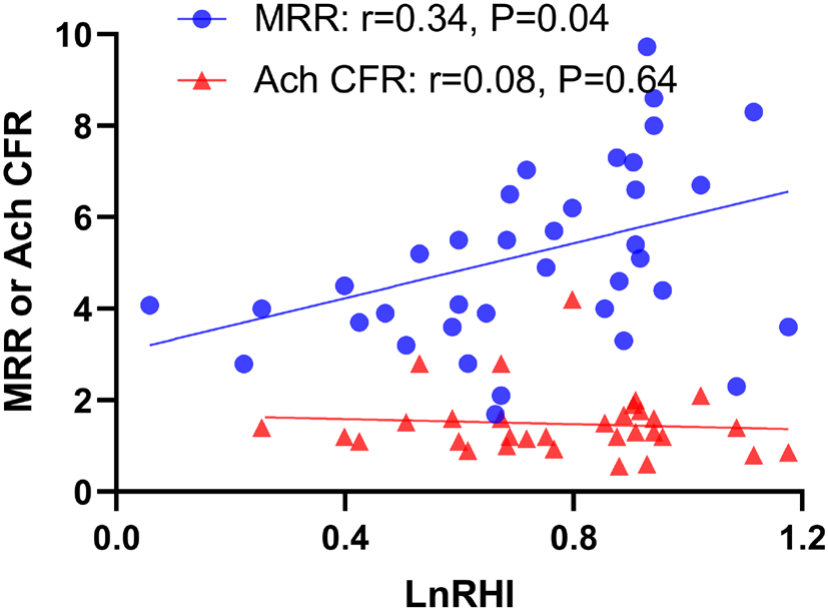
Correlation between the natural logarithm of the reactive hyperemia index (RHI) and coronary microvascular function, assessed by endothelium‐dependent (acetylcholine‐induced coronary flow reserve, Ach CFR) and endothelium‐independent (microvascular resistance reserve, MRR) measures.

**FIGURE 2:**
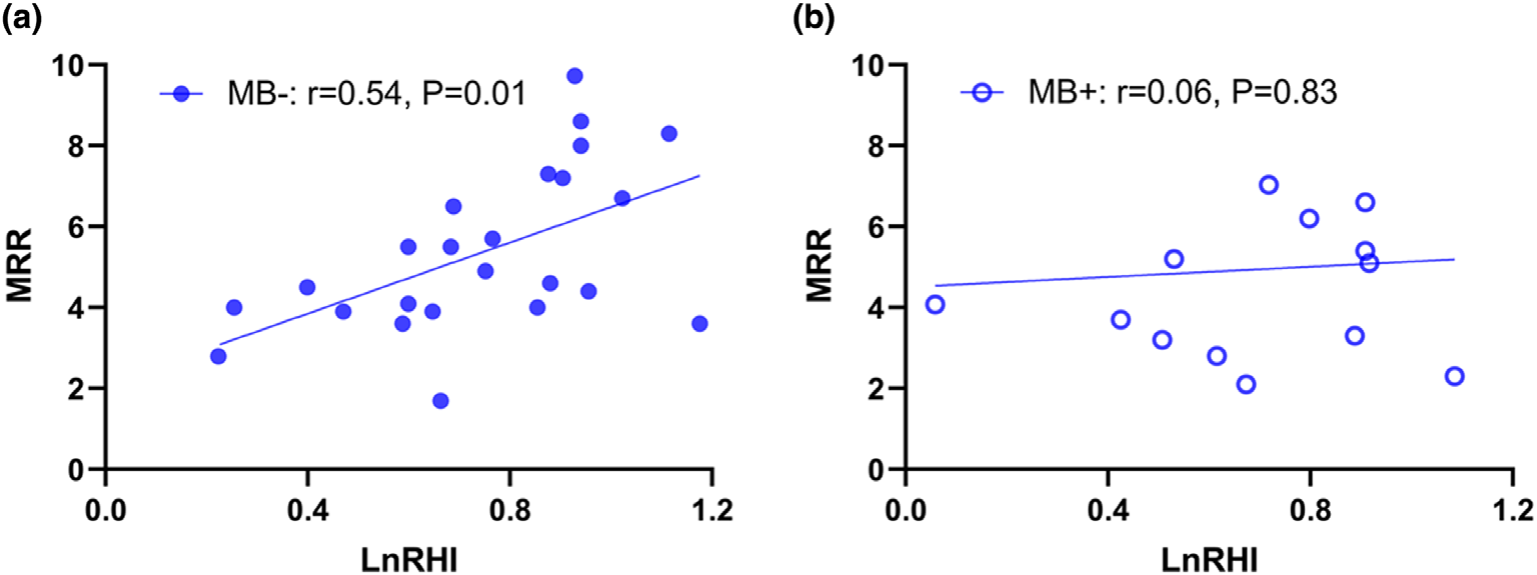
(a) Correlation between the natural logarithm of the reactive hyperemia index (RHI) and endothelium‐independent coronary microvascular function (microvascular resistance reserve, MRR) in patients without myocardial bridging. (b) Correlation between the natural logarithm of RHI and MRR in patients with myocardial bridging.

A significant positive correlation was observed between the natural logarithm of the RHI (LnRHI) and MRR (*r* = 0.34, *p* = 0.04) (Figure 1). However, no significant correlation was found between LnRHI and Ach CFR. Subgroup analysis revealed that the positive correlation between LnRHI and MRR persisted in patients without MB (*N* = 24, *r* = 0.54, *p* = 0.01) but was markedly attenuated in patients with MB (*N* = 14, *p* = 0.83) (Figure 2a,b).

## 4 DISCUSSION

This study identified a significant positive correlation between RHI and endothelium‐independent coronary microvascular function, emphasizing the parallel progression of systemic microvascular dysfunction across different vascular beds. Notably, this correlation was preserved in patients without MB, whereas the association was absent in those with MB, suggesting that MB may act as a localized modifier, disrupting coronary microvascular function independently of systemic microvascular status.

A previous study demonstrated that digital pulse wave amplitude during reactive hyperemia was significantly blunted by inhibiting nitric oxide (NO) synthesis with N(G)‐nitro‐L‐arginine methyl ester (−46 ± 21%; *p* = 0.002). This finding suggests that microvascular function, as assessed by RHI, is approximately 50% dependent on endothelial pathways, with the remaining contribution attributable to endothelium‐independent mechanisms (Nohria et al., 2006). Based on this, we hypothesized that RHI would correlate with both endothelium‐dependent and endothelium‐independent coronary microvascular function. Conversely, another study demonstrated that inwardly rectifying potassium (K_IR) channels and Na^+^/K^+^‐ATPase activation, which induce vascular hyperpolarization, account for nearly 90% of the total reactive hyperemia response in the forearm, whereas NO and prostaglandins play no significant role in this response (Crecelius et al., 2013). Similarly, acetylcholine‐induced coronary vasodilation exhibits a distinct mechanistic dichotomy: in epicardial arteries, vasodilation is predominantly NO‐dependent, whereas in the coronary microvasculature, it is primarily mediated by endothelial hyperpolarization (EDH) via calcium‐activated potassium (K_Ca) channels (Shimokawa & Godo, 2020). Once K_IR channels are activated, they sustain hyperpolarization, facilitating Ca^2+^ influx into endothelial cells via voltage‐sensitive calcium channels. This increase in intracellular Ca^2+^ further enhances K_Ca channel activity, thereby reinforcing the EDH‐mediated vasodilatory response (Jackson, 2022). Given these proposed mechanisms, the precise contributors to the observed reactive hyperemic response remain inconclusive. Interestingly, our study only observed a significant positive correlation between RHI and endothelium‐independent coronary microvascular function. This result contrasts with prior findings showing that patients with endothelium‐dependent coronary microvascular dysfunction had significantly lower RHI compared to those with normal coronary microvascular function (Bonetti et al., 2004). One potential explanation for this discrepancy lies in differences in patient characteristics. The earlier study was conducted in a relatively obese cohort with a mean BMI of 28.2–29.5, whereas our cohort consisted of leaner individuals with a mean BMI of 24.0. Obesity is known to contribute to both coronary microvascular endothelial dysfunction and reduced RHI (Galili et al., 2007; Hamburg et al., 2008; Taher et al., 2019). As such, obesity may act as a confounding factor, strengthening the association between reduced RHI and endothelium‐dependent coronary microvascular dysfunction. Another possible explanation for the lack of correlation between RHI and endothelium‐dependent coronary microvascular function in our study is the role of epicardial adipose tissue (EAT) as a local modifier of coronary endothelial microvascular function. A recent study from the WISE‐CVD (Women's ischemia syndrome evaluation‐coronary vascular dysfunction) reported a negative correlation between magnetic resonance imaging‐derived pericardial fat volume and coronary blood flow response to intracoronary Ach (Landes et al., 2024). Beyond EAT volume, inflammation within EAT has also been implicated in the progression of coronary microvascular dysfunction (Patel et al., 2024). This raises the hypothesis that local modifiers, such as EAT, may interfere with the relationship between RHI and endothelium‐dependent coronary microvascular function. These findings underscore the complexity of microvascular regulation and highlight potential avenues for future research to better elucidate these mechanisms.

The primary hemodynamic consequence of MB is the compression of the coronary artery lumen during systole, which may extend into diastole, thereby altering coronary hemodynamics at the MB site (Ge et al., 1999; Klues et al., 1997). This phenomenon aligns with the conceptual model of a high‐pressure, high‐shear stress environment within the bridged segment (Klues et al., 1997). While moderate shear stress is generally protective for vascular integrity, both excessive and reduced shear stress can disrupt endothelial function, leading to vasomotor dysregulation (Gimbrone et al., 2000; Traub & Berk, 1998). Additionally, elevated intravascular pressure within the bridged segment has been implicated in endothelial dysfunction (Ghaleh et al., 1998; Herrmann & Lerman, 2001; Huang et al., 1998; Ungvari et al., 2003). A case–control study comparing endothelial‐dependent and endothelial‐independent vasomotor function in 29 patients with MB and 58 propensity score‐matched controls without MB demonstrated that endothelium‐dependent vasomotor function was significantly impaired at the MB site relative to both proximal and distal coronary segments, as well as to the control group. Notably, the wall shear rate was markedly elevated in the MB segment, underscoring the intricate interplay between the structural and functional consequences of MB (Herrmann et al., 2004). MB has also been linked to endothelium‐dependent coronary microvascular dysfunction. In a cohort of 1469 INOCA patients undergoing coronary vasomotor function testing, 14.2% had MB, primarily in the LAD (Sara et al., 2020). The presence of MB was associated with higher rates of both epicardial and microvascular endothelial dysfunction, particularly in younger patients (<50 years), suggesting its contribution to ischemia beyond conventional cardiovascular risk factors. Endothelin‐1, a vasoactive mediator known to modulate microvascular tone, may play a key role in this interaction, with its bioavailability exhibiting age‐related variation (Masuda et al., 2001; Miyauchi et al., 1992). Beyond its effects on endothelium‐dependent coronary microvascular function, MB may also influence endothelium‐independent coronary microvascular function. In our study, MRR did not differ between patients with and without MB. However, the correlation between peripheral and coronary microvascular function was lost in patients with MB, supporting the hypothesis that MB acts as a local modifier of coronary microvascular function. Further supporting this hypothesis, the resistive reserve ratio (RRR), a metric more specific for endothelium‐independent microvascular function than CFR, demonstrated superior prognostic value in patients with MB, with a lower RRR associated with worse clinical outcomes (Mahmoudi Hamidabad et al., 2024). These findings highlight the need for a comprehensive assessment of MB‐related alterations in coronary physiology, particularly in the context of microvascular dysfunction.

## 5 LIMITATIONS

We acknowledge several limitations in our study. First, the small sample size of patients with MB from a single center limited the scope for extensive subgroup analyses. As a result, the findings should be interpreted as hypothesis‐generating rather than definitive. Second, MB was identified through visual assessment using coronary angiography without hemodynamic evaluation, which may have led to an underestimation of MB prevalence or severity. Third, the endothelium‐dependent coronary microvascular function was assessed using the thermodilution technique to measure coronary blood flow changes before and after intracoronary Ach administration (Miner et al., 2023). While the gold standard involves Doppler wire‐based measurement of coronary blood flow changes, as performed in specialized centers, Doppler wire was unavailable in our region (Kanaji et al., 2024; Sinha et al., 2024). However, prior studies have demonstrated a strong correlation between Doppler‐measured and thermodilution‐measured coronary blood flow changes, supporting the validity of our approach (Melikian et al., 2007). Finally, we utilized RHI to assess microvascular function. However, prior studies have indicated that RHI and FMD capture distinct aspects of vascular physiology and are associated with different atherosclerotic risk factors (Hamburg et al., 2011). Although both methodologies evaluate vascular function via reactive hyperemia, they are believed to reflect distinct physiological mechanisms (Schnabel et al., 2011). Furthermore, a study demonstrated that while FMD is significantly impaired with aging, RHI does not exhibit a comparable age‐related decline, potentially explaining the lack of correlation between these two indices (Babcock et al., 2021). Given the well‐established role of NO in FMD, these findings suggest that RHI may be less reflective of endothelial function, aligning with our observation of no significant correlation between RHI and coronary microvascular endothelial function. Despite these limitations, our findings provide valuable insights into the relationship between peripheral and coronary microvascular function, as well as the potential impact of MB on coronary microvascular physiology.

## 6 CONCLUSIONS

Peripheral microvascular function gauged by RHI was positively correlated with endothelium‐independent coronary microvascular function in patients without MB. MB may potentially affect coronary microvascular function, leading to the loss of association between peripheral and coronary microvascular function.

## AUTHOR CONTRIBUTIONS

TT conceived and designed the study, prepared the figures, and drafted the manuscript. TT and SY performed data analysis and interpreted the results. KK, SA, TU, KN, MO, YN, AM, YN and TA critically reviewed, edited, and revised the manuscript. All authors have read and approved the final version of the manuscript.

## FUNDING INFORMATION

None.

## CONFLICT OF INTEREST STATEMENT

The authors declare that the research was conducted in the absence of any commercial or financial relationships that could be construed as a potential conflict of interest.

## ETHICS STATEMENT

The study adhered to the principles outlined in the Declaration of Helsinki and received approval from the National Defense Medical College ethics committee (#4803). Written informed consent was obtained from all participants prior to their inclusion in the study.

## Data Availability

The data supporting the findings of this study are available from the corresponding authors upon reasonable request.
